# A systematic review and meta‐analysis of the association between the D‐dimer and rheumatic diseases

**DOI:** 10.1002/iid3.1349

**Published:** 2024-07-26

**Authors:** Angelo Zinellu, Arduino A. Mangoni

**Affiliations:** ^1^ Department of Biomedical Sciences University of Sassari Sassari Italy; ^2^ Discipline of Clinical Pharmacology, College of Medicine and Public Health Flinders University Adelaide Australia; ^3^ Department of Clinical Pharmacology Flinders Medical Centre, Southern Adelaide Local Health Network Adelaide Australia

**Keywords:** autoimmunity, d‐dimer, disease activity, hypercoagulability, inflammation, rheumatic diseases, venous thromboembolism

## Abstract

**Introduction:**

There is good evidence that specific autoimmune rheumatic diseases (RDs), for example, rheumatoid arthritis and systemic lupus erythematosus (SLE), are associated with a state of hypercoagulability and an increased risk of venous thromboembolism (VTE). However, limited information regarding this association is available for other autoimmune or autoinflammatory RDs. We sought to address this issue by conducting a systematic review and meta‐analysis of the association between the d‐dimer, an established marker of hypercoagulability and VTE, and RDs and the possible clinical and demographic factors mediating this association.

**Methods:**

We searched the electronic databases PubMed, Web of Science, and Scopus from inception to January 31, 2024. The risk of bias and the certainty of evidence were assessed using the Joanna Briggs Institute Critical Appraisal Checklist and GRADE, respectively.

**Results:**

In 31 studies selected for analysis (2724 RD patients and 3437 healthy controls), RD patients had overall significantly higher d‐dimer concentrations when compared to controls (standard mean difference = 0.93, 95% CI 0.76−1.10, *p* < .001; *I* ^2^ = 86.1%, *p* < .001; moderate certainty of evidence). The results were stable in a sensitivity analysis. Significant associations were observed between the effect size of the between‐group differences in d‐dimer concentration and age, specific RD and RD category, RD duration, fibrinogen, plasminogen activator inhibitor, C‐reactive protein, and erythrocyte sedimentation rate.

**Conclusions:**

Overall, patients with RDs have significantly higher d‐dimer concentrations when compared with healthy controls, indicating a state of hypercoagulability. The alterations in d‐dimer concentrations are mediated by age, specific RD and RD category, RD duration, and markers of anticoagulation and inflammation. Further research is warranted to investigate d‐dimer concentrations across the spectrum of RDs and their utility in predicting and managing VTE in these patients (PROSPERO registration number: CRD42024517712).

## Graphical abstract

Our systematic review and meta‐analysis have shown that, overall, patients with rheumatic diseases (RDs) have significantly higher d‐dimer concentrations when compared with healthy controls, indicating a state of hypercoagulability. The alterations in d‐dimer concentrations are mediated by age, specific RD and RD category, RD duration, and markers of anticoagulation and inflammation.

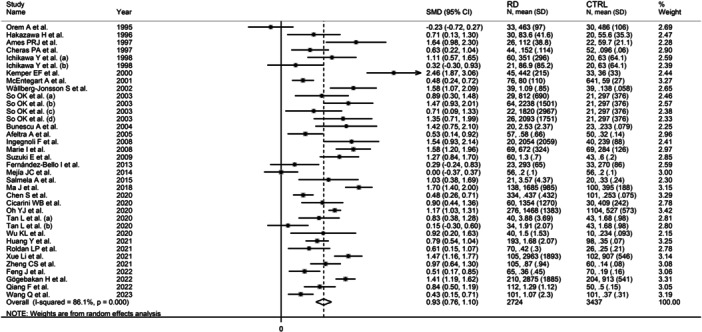

## 1 INTRODUCTION

Rheumatic diseases (RDs) encompass a wide range of chronic conditions with a predominantly autoimmune (e.g., rheumatoid arthritis, RA), a mixed‐autoimmune‐autoinflammatory (e.g., ankylosing spondylitis), or an autoinflammatory component (e.g., familial Mediterranean fever).^1^, ^2^, ^3^ Regardless of this broad categorization, individual RDs are generally characterized by disabling symptoms, significant complications, and overall poor quality of life despite the availability of safe and effective pharmacological and nonpharmacological treatment strategies.^4^, ^5^, ^6^, ^7^, ^8^, ^9^, ^10^, ^11^, ^12^, ^13^, ^14^, ^15^, ^16^ One important factor contributing to the health burden of RDs on patients and healthcare systems is represented by a state of hypercoagulability with a predisposition to venous thromboembolism (VTE).^17^, ^18^ This issue has been well studied in specific RDs, for example, RA (increased risk of VTE by a factor of 2−2.5 vs. general population),^19^, ^20^, ^21^ systemic lupus erythematosus (SLE, increased risk of VTE by a factor of 4.38 vs. general population),^22^, ^23^ systemic sclerosis (SSc) (increased risk of VTE by a factor of 2.5 vs. general population),^24^ ANCA‐associated vasculitis (AAV) (increased risk of VTE by a factor of 3.26 vs. general population),^25^, ^26^, ^27^, ^28^, ^29^, ^30^, ^31^ CA and gout (increased risk of VTE by a factor of 1.33 vs. general population),^32^ osteoarthritis (OA) (increased risk of VTE by a factor of 1.38 vs. general population),^33^ and Behcet disease (BD) (increased risk of VTE by a factor of 2.80 vs. general population).^34^, ^35^, ^36^, ^37^

It is commonly accepted that the proinflammatory and pro‐oxidant state in patients with RA and SLE favors the upregulation of procoagulant pathways and the downregulation of anticoagulant and fibrinolytic pathways.^18^, ^38^, ^39^, ^40^ In support of this theory, several epidemiological and experimental studies have reported a higher tendency to coagulation and VTE in RA and SLE patients with active disease versus those in remission.^41^, ^42^, ^43^ However, the mechanisms underpinning the complex interplay between inflammation, oxidative stress, coagulation, and thrombosis have been less studied in other RDs, particularly those with a mixed‐autoimmune‐autoinflammatory or autoinflammatory component.

The d‐dimer is one of the main degradation products of fibrin. It is generated following the cleavage of crosslinked fibrin by plasmin and consists of two D domains from adjacent fibrin monomers crosslinked by activated factor XIII.^44^, ^45^ d‐dimer concentrations are routinely measured when suspecting a state of hypercoagulability and as part of the clinical assessment to determine the probability of VTE.^46^, ^47^, ^48^ In the context of RDs, although the clinical significance of the d‐dimer has been primarily investigated in patients with RA,^49^, ^50^, ^51^ an increasing number of studies has assessed the pathophysiological role of this coagulation biomarker in other autoimmune and autoinflammatory conditions.

Therefore, we critically appraised the available evidence regarding the association between the d‐dimer and RDs by conducting a systematic review and meta‐analysis of studies assessing d‐dimer concentrations in patients with different RDs and healthy controls. Furthermore, we conducted a series of meta‐regression and subgroup analyses to identify possible clinical and demographic factors mediating the association between the d‐dimer and RDs.

## 2 MATERIALS AND METHODS

### 2.1 Search strategy and study selection

We systematically searched electronic databases (PubMed, Web of Science, and Scopus) from inception to January 31, 2024, for relevant articles using the following terms: “d‐dimer” AND “rheumatic diseases” OR “rheumatoid arthritis” OR “psoriatic arthritis” OR “reactive arthritis” OR “ankylosing spondylitis” OR “systemic lupus erythematosus” OR “systemic sclerosis” OR “scleroderma” OR “Sjogren's syndrome” OR “connective tissue diseases” OR “vasculitis” OR “Behçet's disease” OR “idiopathic inflammatory myositis” OR “polymyositis” OR “dermatomyositis” OR “gout” OR “pseudogout” OR “systemic vasculitis” OR “ANCA‐associated vasculitis” OR “Takayasu arteritis” OR “polyarteritis nodosa” OR “osteoarthritis” OR “fibromyalgia” OR “granulomatous polyangiitis” OR “Henoch‐Schonlein purpura” OR “Wegener's granulomatosis” OR “familial Mediterranean fever.” Two investigators independently screened abstracts and full‐text articles according to the following inclusion criteria: (i) assessment of d‐dimer, (ii) comparison between RD patients and healthy controls in case‐control studies, (iii) use of English language, and (iv) availability of the full‐text of the article. The references of each article were hand‐searched for additional articles.

The two investigators independently extracted the following variables for analysis: publication year, first author details, the country where the study was conducted, type of RD, d‐dimer concentrations, number of participants, age, male‐to‐female ratio, mean RD duration, fibrinogen, C‐reactive protein (CRP), erythrocyte sedimentation rate (ESR), tissue plasminogen activator (t‐PA), plasminogen activator inhibitor (PAI‐1), and use of glucocorticoids and disease‐modifying anti‐rheumatic drugs (DMARDs).

We assessed the risk of bias using the Joanna Briggs Institute Critical Appraisal Checklist for analytical studies, which considers the following domains: clear definition of inclusion criteria, detailed description of participants and setting, reliable measurement of the exposure, use of standard criteria to assess the condition, identification, and management of confounding factors, reliable measurement of the outcome, and appropriate use of statistical analysis.^52^ The risk was considered high, intermediate, or low for studies that addressed <50%, ≥50% and <75%, and ≥75% of checklist items. The certainty of evidence was assessed using the GRADE system.^53^ We complied with the PRISMA 2020 statement (Table S1),^54^ and registered our protocol in an international repository (PROSPERO registration number: CRD42024517712).

### 2.2 Statistical analysis

We generated forest plots of standardized mean differences (SMDs) and 95% confidence intervals (CIs) to assess differences in d‐dimer concentrations between RD patients and healthy controls (a *p* < .05 was considered statistically significant). A positive pooled SMD value indicated higher d‐dimer concentrations in RD patients compared to controls. By contrast, a negative pooled SMD value indicated lower d‐dimer concentrations in RD patients compared to controls. If necessary, means and standard deviations were extrapolated using accepted methods.^55^ The *Q* statistic was used to assess the heterogeneity of the SMD across studies (a *p* < .01 was considered statistically significant). A random‐effect model based on the inverse‐variance method was used if high heterogeneity was present.^56^, ^57^ Sensitivity analysis and assessment of publication bias were performed according to standard procedures.^58^, ^59^, ^60^, ^61^ We conducted meta‐regression and subgroup analyses to investigate associations between the effect size and the following parameters: year of publication, the geographical area where the study was conducted, RD type and RD category, sample size, age, male‐to‐female ratio, mean RD duration, fibrinogen, CRP, ESR, t‐PA, PAI‐1, and use of glucocorticoids and DMARDs. Statistical analyses were performed using Stata 14 (Stata Corp.).

## 3 RESULTS

**Figure 1:**
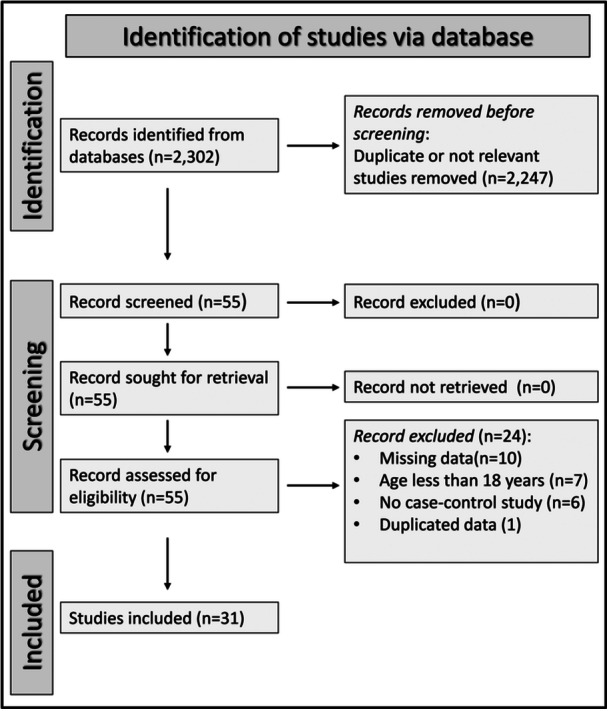
PRISMA 2020 flow diagram.

Our search criteria identified 2302 articles, of which 2247 were excluded because of irrelevance or duplication. After a full‐text assessment of the remaining 55 articles, 10 were excluded because they did not report critical information, seven because they recruited non‐adult participants, six because they had a different study design, and one because it presented data that duplicated those of another study. Therefore, 31 studies were selected for analysis^62^, ^63^, ^64^, ^65^, ^66^, ^67^, ^68^, ^69^, ^70^, ^71^, ^72^, ^73^, ^74^, ^75^, ^76^, ^77^, ^78^, ^79^, ^80^, ^81^, ^82^, ^83^, ^84^, ^85^, ^86^, ^87^, ^88^, ^89^, ^90^, ^91^, ^92^ (Figure 1 and Table 1). The risk of bias was ranked as low in 20 studies^70^, ^71^, ^74^, ^76^, ^77^, ^78^, ^79^, ^80^, ^81^, ^82^, ^83^, ^84^, ^85^, ^86^, ^87^, ^88^, ^89^, ^90^, ^91^, ^92^ and moderate in the remaining 11^62^, ^63^, ^64^, ^65^, ^66^, ^67^, ^68^, ^69^, ^72^, ^73^, ^75^ (Table 2). The cross‐sectional nature of the studies selected downgraded the initial level of certainty to low.

**Table 1:** Characteristics of the studies investigating d‐dimer concentrations in rheumatic diseases.

Study	Healthy controls				Patients with rheumatic diseases				Disease type	MDD (years)	Design
	*n*	Age (years)	M/F	d‐dimer (mean ± SD)	*n*	Age (years)	M/F	d‐dimer (mean ± SD)			
Orem et al. 1995, Turkey^62^	30	32	15/15	486 ± 106	33	29	19/14	463 ± 97	BD	NR	P
Akazawa et al. 1996, Japan^63^	20	44	1/19	55.6 ± 35.5	30	45	1/29	83.6 ± 41.6	TA	13	P
Ames et al. 1997, Portugal^64^	22	37	2/20	59.7 ± 21.1	26	41	3/23	112 ± 38.8	SSc	8.4	P
Cheras et al. 1997, Australia^65^	52	68	31/21	0.096 ± 0.06	44	69	26/18	0.152 ± 0.114	OA	NR	P
Ichikawa et al. 1998, Japan^66^	20	57	0/20	63 ± 64.1	60	56	7/53	351.2 ± 296.3	RA	11.5	P
Ichikawa et al. 1998, Japan^66^	20	57	0/20	63 ± 64.1	21	38	3/18	86.9 ± 85.2	SLE	11.7	P
Kamper et al. 2000, Greece^67^	33	53	10/23	36 ± 33	45	60	12/33	442 ± 215	RA	4.9	P
McEntegart et al. 2001, UK^68^	641	NR	NR	59 ± 27	76	NR	13/63	80 ± 110	RA	12.5	P
Wållberg‐Jonsson et al. 2002, Sweden^69^	39	Matched	Matched	0.138 ± 0.058	39	52	9/30	1.088 ± 0.85	RA	NR	P
So et al. 2003, Germany^70^	21	45	9/12	297 ± 376	29	67	11/18	812 ± 690	OA	NR	P
So et al. 2003, Germany^70^	21	45	9/12	297 ± 376	64	55	15/49	2238 ± 1501	RA	NR	P
So et al. 2003, Germany^70^	21	45	9/12	297 ± 376	22	38	14/8	1820 ± 2967	SpA	NR	P
So et al. 2003, Germany^70^	21	45	9/12	297 ± 376	26	72	14/12	2093 ± 1751	CA	NR	P
Bunescu et al. 2004, Sweden^71^	23	55	5/18	0.233 ± 0.079	20	56	5/15	2.53 ± 2.37	RA	NR	P
Afeltra et al. 2005, Italy^72^	50	Matched	Matched	0.32 ± 0.14	57	40	8/49	0.58 ± 0.66	SLE	9.9	R
Ingegnoli et al. 2008, Italy^73^	40	Matched	Matched	239 ± 88	20	55	5/15	2054 ± 2059	RA	6.1	P
Marie et al. 2008, France^74^	69	Matched	Matched	284 ± 126	69	58	9/60	672 ± 324	SSc	NR	P
Suzuki et al. 2009, Japan^75^	43	28	18/25	0.6 ± 0.2	60	45	3/57	1.3 ± 0.7	SLE	15.5	P
Fernández‐Bello et al. 2013, Spain^76^	33	43	12/21	270 ± 86	23	49	5/18	293 ± 65	BD	15	P
Mejía et al. 2014, Spain^77^	56	35	30/26	0.2 ± 0.1	56	34	30/26	0.2 ± 0.1	BD	NR	P
Salmela et al. 2015, Finland^78^	20	58	14/6	0.33 ± 0.24	21	60	16/5	3.57 ± 4.37	AAV	NR	P
Ma et al. 2018, China^79^	100	35	50/50	395 ± 188	138	65	45/93	1685 ± 985	RA	NR	P
Chen et al. 2020, China^80^	101	37	5/96	0.253 ± 0.075	334	40	9/325	0.437 ± 0.432	Gout	NR	R
Cicarini et al. 2020, Brazil^81^	30	NR	0/30	409 ± 242	60	40	0/60	1354 ± 1270	SLE	8.5	P
Oh et al. 2020, Korea^82^	1104	38	172/932	527 ± 573	276	37	35/241	1468 ± 1383	SLE	8.4	R
Tan et al. 2020, China^83^	43	67	22/21	1.68 ± 0.98	40	67	23/17	3.88 ± 3.69	AAV	NR	P
Tan et al. 2020, China^83^	43	67	22/21	1.68 ± 0.98	34	62	14/20	1.91 ± 2.07	SLE	NR	P
Wu et al. 2020, China^84^	10	56	5/5	0.234 ± 0.093	40	58	19/21	1.5 ± 1.53	AAV	NR	P
Huang et al. 2021, China^86^	98	37	20/78	0.35 ± 0.07	193	39	10/183	1.68 ± 2.07	SLE	NR	R
Roldan et al. 2021, USA^87^	26	32	4/22	0.25 ± 0.21	70	36	6/64	0.42 ± 0.3	SLE	8	P
Xue et al. 2021, China^88^	102	54	26/76	907 ± 546	105	55	27/78	2963 ± 1893	RA	NR	R
Zheng et al. 2021, China^89^	60	39	6/54	0.14 ± 0.08	105	42	10/95	0.87 ± 0.94	SLE	NR	R
Feng et al. 2022, China^85^	70	34	59/11	0.19 ± 0.16	65	26	61/4	0.36 ± 0.45	SpA	NR	P
Gögebakan et al. 2022, Turkey^90^	204	34	152/52	913 ± 541	210	35	156/54	2875 ± 1885	AS	9.4	R
Qiang et al. 2022, China^91^	50	NR	NR	0.5 ± 0.15	112	55	24/88	1.29 ± 1.12	RA	NR	P
Wang et al. 2022, China^92^	101	54	2/99	0.37 ± 0.31	101	54	2/99	1.07 ± 2.3	pSS	NR	P

*Note*: d‐dimer serum concentrations are expressed as ng/mL, µg/mL, mg/dL, or g/mL.

Abbreviations: AAV, ANCA‐associated vasculitis; AS, ankylosing spondylitis; BD, Behcet disease; CA, crystal arthritis; MDD, mean disease duration; NR, not reported; OA, osteoarthritis; P, prospective; pSS, primary Sjögren's Syndrome; R, retrospective; RA, rheumatoid arthritis; SLE, systemic lupus erythematosus; SpA, spondyloarthritis; SSc, systemic sclerosis; TA, Takayasu arteritis.

**Table 2:** Assessment of the risk of bias using the Joanna Briggs Institute critical appraisal checklist.

Study	Were the inclusion criteria clearly defined?	Were the subjects and the setting described in detail?	Was the exposure measured in a reliable way?	Were standard criteria used to assess the condition?	Were confounding factors identified?	Were strategies to deal with confounding factors stated?	Were the outcomes measured in a reliable way?	Was appropriate statistical analysis used?	Risk of bias
Orem et al.^62^	No	Yes	Yes	Yes	No	No	Yes	Yes	Moderate
Akazawa et al.^63^	No	Yes	Yes	Yes	No	No	Yes	Yes	Moderate
Ames et al.^64^	No	Yes	Yes	Yes	No	No	Yes	Yes	Moderate
Cheras et al.^65^	No	Yes	Yes	Yes	No	No	Yes	Yes	Moderate
Ichikawa et al.^66^	No	Yes	Yes	Yes	No	No	Yes	Yes	Moderate
Kamper et al.^67^	No	Yes	Yes	Yes	No	No	Yes	Yes	Moderate
McEntegart et al.^68^	No	Yes	Yes	Yes	No	No	Yes	Yes	Moderate
Wållberg‐Jonsson et al.^69^	No	Yes	Yes	Yes	No	No	Yes	Yes	Moderate
So et al.^70^	Yes	Yes	Yes	Yes	No	No	Yes	Yes	Low
Bunescu et al.^71^	Yes	Yes	Yes	Yes	No	No	Yes	Yes	Low
Afeltra et al.^72^	No	Yes	Yes	Yes	No	No	Yes	Yes	Moderate
Ingegnoli et al.^73^	No	Yes	Yes	Yes	No	No	Yes	Yes	Moderate
Marie et al.^74^	Yes	Yes	Yes	Yes	No	No	Yes	Yes	Low
Suzuki et al.^75^	No	Yes	Yes	Yes	No	No	Yes	Yes	Moderate
Fernández‐Bello et al.^76^	Yes	Yes	Yes	Yes	No	No	Yes	Yes	Low
Mejía et al.^77^	Yes	Yes	Yes	Yes	No	No	Yes	Yes	Low
Salmela et al.^78^	Yes	Yes	Yes	Yes	No	No	Yes	Yes	Low
Ma et al.^79^	Yes	Yes	Yes	Yes	No	No	Yes	Yes	Low
Chen et al.^80^	Yes	Yes	Yes	Yes	Yes	Yes	Yes	Yes	Low
Cicarini et al.^81^	Yes	Yes	Yes	Yes	Yes	Yes	Yes	Yes	Low
Oh et al.^82^	No	Yes	Yes	Yes	Yes	Yes	Yes	Yes	Low
Tan et al.^83^	Yes	Yes	Yes	Yes	Yes	Yes	Yes	Yes	Low
Wu et al.^84^	Yes	Yes	Yes	Yes	No	No	Yes	Yes	Low
Huang et al.^86^	Yes	Yes	Yes	Yes	No	No	Yes	Yes	Low
Roldan et al.^87^	Yes	Yes	Yes	Yes	No	No	Yes	Yes	Low
Xue et al.^88^	Yes	Yes	Yes	Yes	Yes	Yes	Yes	Yes	Low
Zheng et al.^89^	Yes	Yes	Yes	Yes	No	No	Yes	Yes	Low
Feng et al.^85^	Yes	Yes	Yes	Yes	Yes	Yes	Yes	Yes	Low
Gögebakan et al.^90^	Yes	Yes	Yes	Yes	Yes	Yes	Yes	Yes	Low
Qiang et al.^91^	Yes	Yes	Yes	Yes	No	No	Yes	Yes	Low
Wang et al.^92^	Yes	Yes	Yes	Yes	No	No	Yes	Yes	Low

The 31 selected studies, including a total of 36 group comparators, assessed the d‐dimer in 2724 RD patients (mean age 46 years, 75% females) and 3437 healthy controls (mean age 42 years, 72% females). The continents where the studies were conducted included Asia (*n* = 16),^62^, ^63^, ^66^, ^75^, ^79^, ^80^, ^82^, ^83^, ^84^, ^85^, ^86^, ^88^, ^89^, ^90^, ^91^, ^92^ Europe (*n* = 12),^64^, ^67^, ^68^, ^69^, ^70^, ^71^, ^72^, ^73^, ^74^, ^76^, ^77^, ^78^ America (*n* = 2),^81^, ^87^ and Oceania (*n* = 1).^65^ RA was investigated in 10 study groups,^66^, ^67^, ^68^, ^69^, ^70^, ^71^, ^73^, ^79^, ^88^, ^91^ SLE in nine,^66^, ^72^, ^75^, ^81^, ^82^, ^83^, ^86^, ^87^, ^89^ BD in three,^62^, ^76^, ^77^ AAV in three,^78^, ^83^, ^84^ spondyloarthritis (SpA) and AS in three,^70^, ^85^, ^90^ SSc in two,^64^, ^74^ OA in two,^65^, ^70^ gout in one,^80^ crystal arthritis in one,^70^ Takayasu arteritis (TA) in one,^63^ and primary Sjögren's Syndrome (pSS) in one.^92^ Twenty‐four studies were prospective^62^, ^63^, ^64^, ^65^, ^66^, ^67^, ^68^, ^69^, ^70^, ^71^, ^73^, ^74^, ^75^, ^76^, ^77^, ^78^, ^79^, ^81^, ^83^, ^84^, ^85^, ^87^, ^91^, ^92^ and the remaining seven retrospective.^72^, ^80^, ^82^, ^86^, ^88^, ^89^, ^90^ Mean disease duration, reported in 14 study groups, ranged between 4.9 and 15 years.^63^, ^64^, ^66^, ^67^, ^68^, ^72^, ^73^, ^75^, ^76^, ^81^, ^82^, ^87^, ^90^ Only three studies reported the use of duplex ultrasonography and/or venography^72^, ^77^, ^82^ and six reported the percentage of subjects with deep vein thrombosis or VTE.^71^, ^72^, ^74^, ^77^, ^78^, ^82^ However, this information was not further analyzed in these studies.

**Figure 2:**
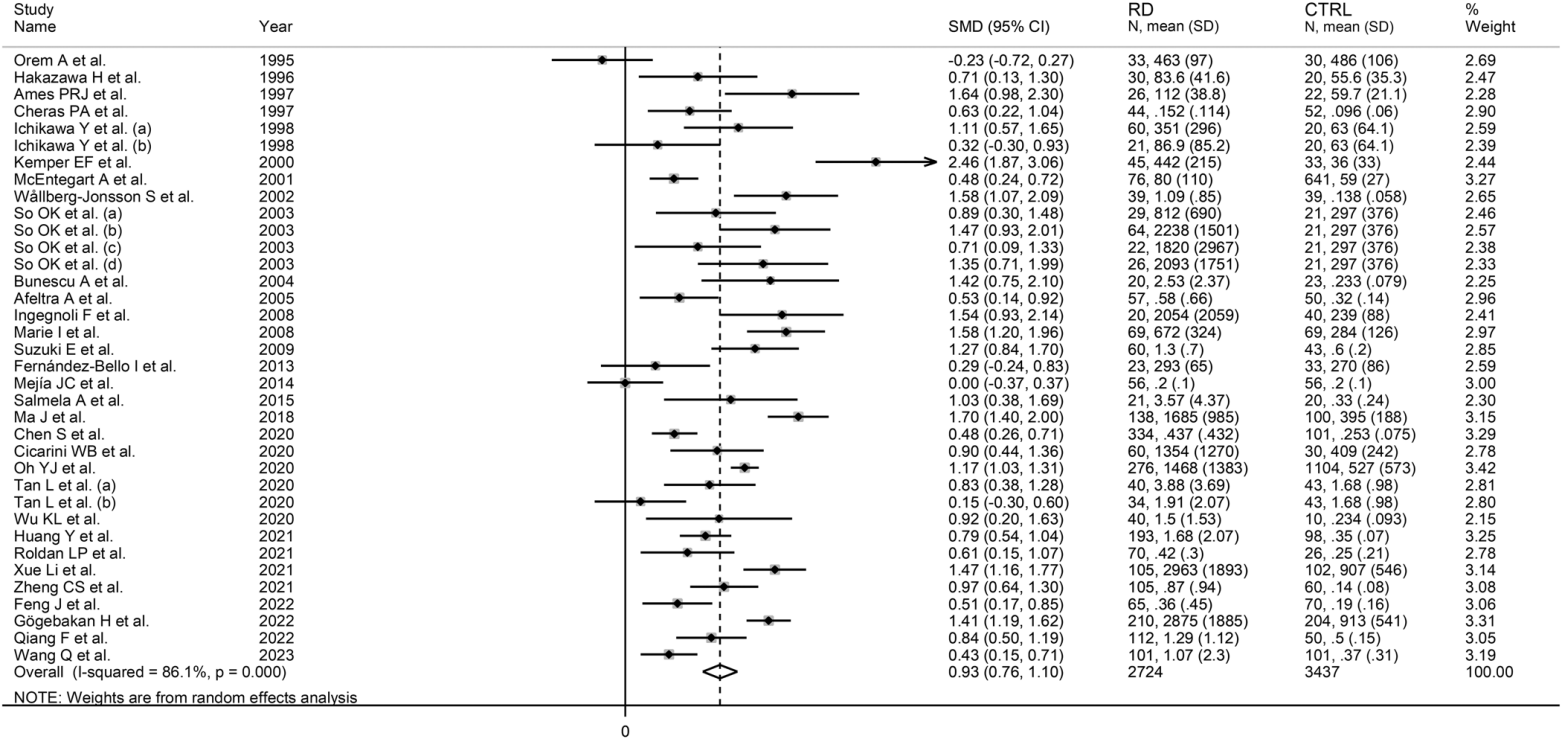
Forest plot of studies investigating d‐dimer concentrations in patients with rheumatic diseases and healthy controls.

**Figure 3:**
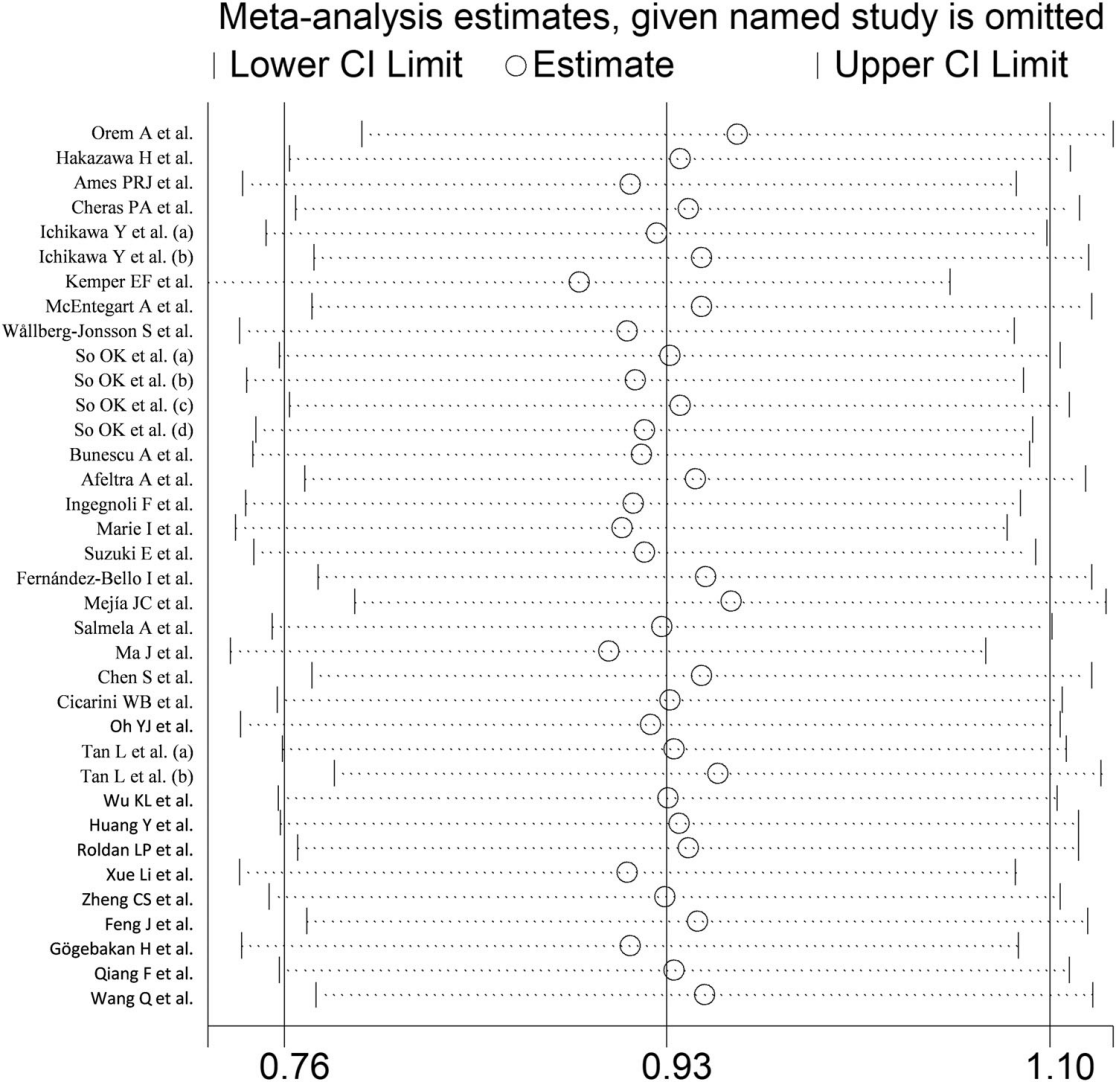
Sensitivity analysis of the association between the d‐dimer and rheumatic diseases.

The forest plot showed that, overall, RD patients had significantly higher d‐dimer concentrations when compared to controls (SMD = 0.93, 95% CI 0.76−1.10, *p* < .001; *I* ^2^ = 86.1%, *p* < .001; Figure 2), with stable corresponding pooled SMD in the sensitivity analysis (range 0.89−0.96, Figure 3).

**Figure 4:**
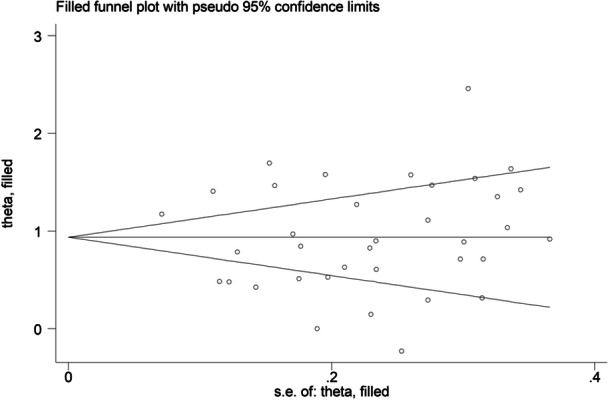
Funnel plot of studies investigating the association between d‐dimer concentrations and rheumatic diseases after “trimming‐and‐filling.”

Neither Begg's test (*p* = .32), Egger's test (*p* = .79), nor the “trim‐and‐fill” method showed significant publication bias (Figure 4).

**Figure 5:**
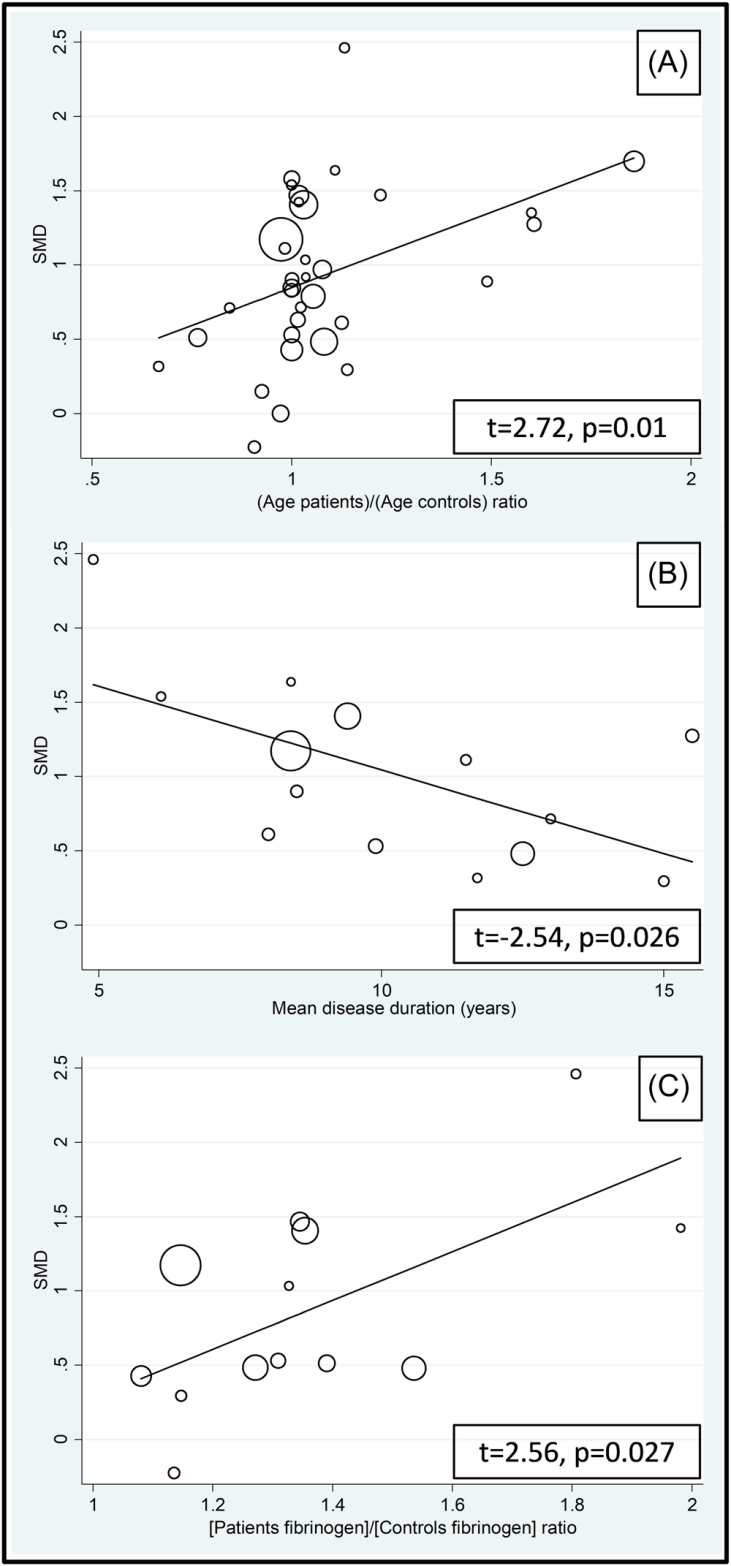
Bubble plot of the univariate meta‐regression analysis between the effect size and age (A), mean disease duration (B), and fibrinogen (C).

**Figure 6:**
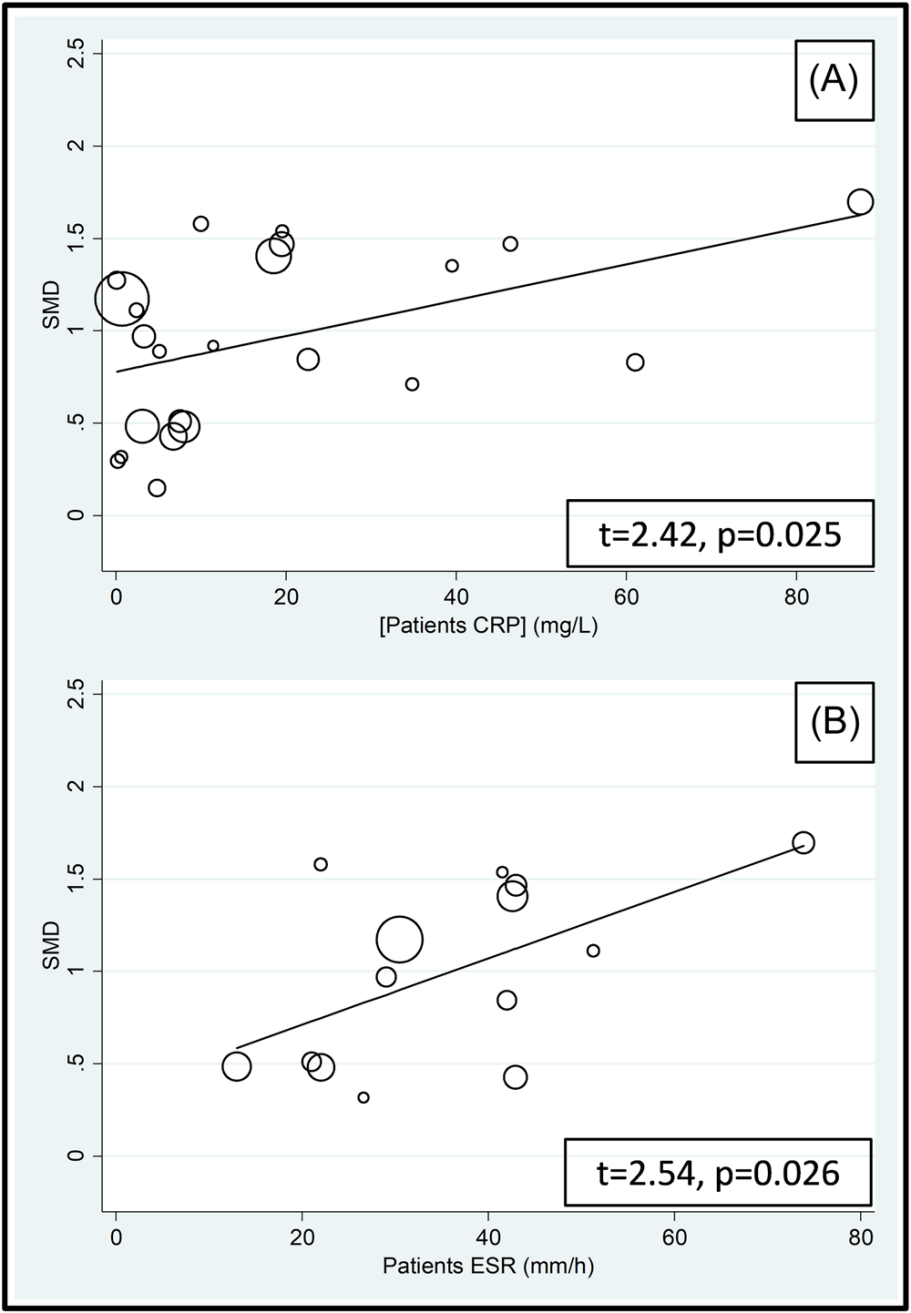
Bubble plot of the univariate meta‐regression analysis between the effect size and C‐reactive protein (CRP) (A) and erythrocyte sedimentation rate (ESR) (B).

The effect size was not associated with the male‐to‐female ratio, sample size, publication year, or use of glucocorticoids and DMARDs. By contrast, significant associations were observed with age, mean RD duration, fibrinogen, CRP, and ESR (Figures 5 and 6; Table 3).

**Table 3:** Meta‐regression analysis to evaluate the association between study and patient characteristics and the SMD.

	*t*‐value	*p* Value
Age	2.72	.01
Male‐to‐female ratio	0.69	.50
Sample size	0.14	.89
Publication year	−0.53	.60
Use of glucocorticoids	−1.83	.10
Fibrinogen	2.56	.027
CRP	2.42	.025
ESR	2.54	.026
Use of DMARDS	1.34	.22
Mean RD duration	−2.54	.026

Abbreviations: CRP, C‐reactive protein; DMARD, disease‐modifying anti‐rheumatic drugs; ESR, erythrocyte sedimentation rate; SMD, standard mean difference.

**Figure 7:**
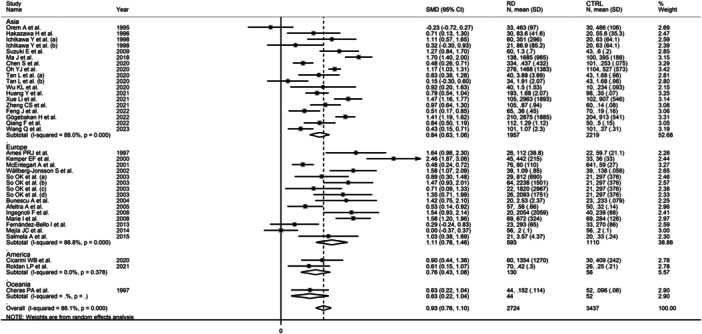
Forest plot of studies investigating d‐dimer concentrations in patients with rheumatic diseases and healthy controls according to country where the study was conducted.

There were nonsignificant differences (*p* = .76) in the pooled SMD between studies conducted in Asia (SMD = 0.84, 95% CI 0.63−1.06, *p* < .001; *I* ^2^ = 88.0%, p < .001), Europe (SMD = 1.11, 95% CI 0.76−1.46, *p* < .001; *I* ^2^ = 86.8%, *p* < .001), and America (SMD = 0.76, 95% CI 0.43−1.08, *p* < .001; *I* ^2^ = 0.0%, *p* = .378, Figure 7), with a virtually absent heterogeneity in the American subgroup.

**Figure 8:**
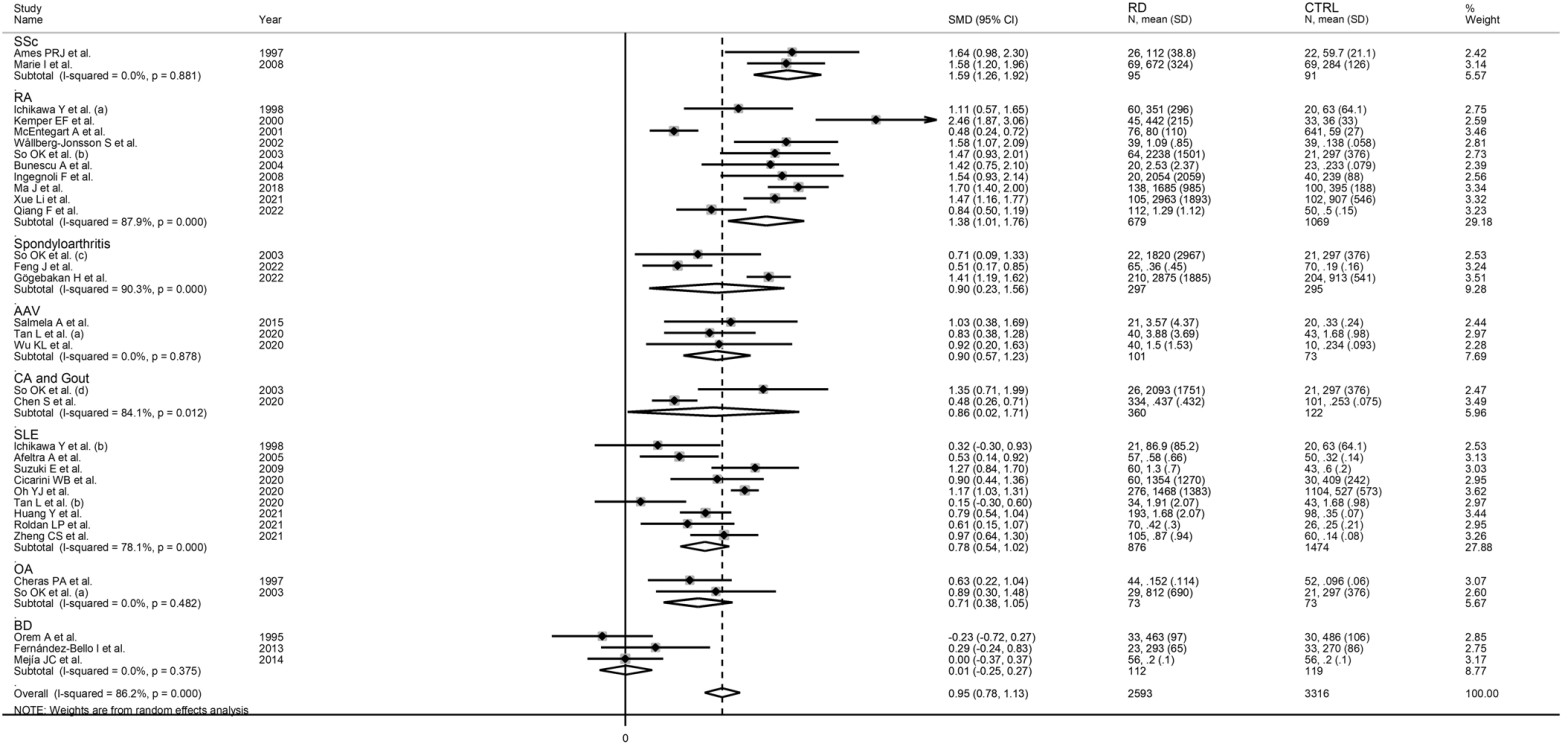
Forest plot of studies investigating d‐dimer concentrations in patients with rheumatic diseases and healthy controls according to specific types of rheumatic disease.

By contrast, there was a significant difference (*p* < .001) in the pooled SMD among different RDs, which progressively decreased in SSc (SMD = 1.59, 95% CI 1.26−1.92, *p* < .001; *I* ^2^ = 0.0%, *p* = .88), RA (SMD = 1.38, 95% CI 1.01−1.76, *p* < .001; *I* ^2^ = 87.9%, *p* < .001), SpA (SMD = 0.90, 95% CI 0.23−1.56, *p* = .008; *I* ^2^ = 90.3%, *p* < .001), AAV (SMD = 0.90, 95% CI 0.57−1.23, *p* < .001; *I* ^2^ = 0.0%, *p* = .878), CA and gout (SMD = 0.86, 95% CI 0.02−1.71, *p* = .045; *I* ^2^ = 84.1%, *p* = .012), SLE (SMD = 0.78, 95% CI 0.54−1.02, *p* < .001; *I* ^2^ = 78.1%, *p* < .001), OA (SMD = 0.71, 95% CI 0.38−1.05, *p* < .001; *I* ^2^ = 0.0%, *p* = .482), and BD (SMD = 0.01, 95% CI −0.25 to 0.27, *p* = .96; *I* ^2^ = 0.0%, *p* = .375, Figure 8) with a virtually absent heterogeneity in the SSc, AAV, OA, and BD subgroups (the SMD in the BD subgroup was not statistically significant).

**Figure 9:**
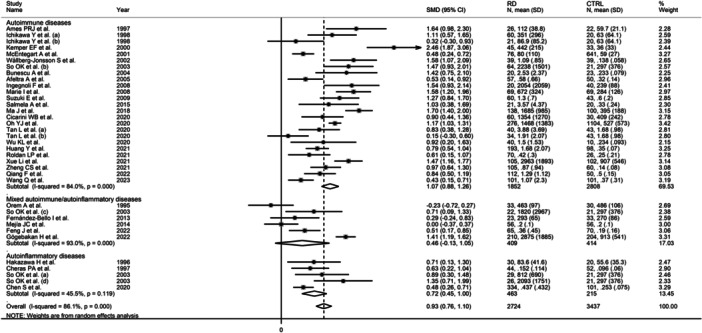
Forest plot of studies investigating d‐dimer concentrations in patients with rheumatic diseases and healthy controls according to the category of rheumatic diseases.

Furthermore, the pooled SMD was significant in studies conducted in patients with autoimmune (SMD = 1.07, 95% CI 0.88−1.26, *p* < .001; *I* ^2^ = 84.0%, *p* < .001) and autoinflammatory diseases (SMD = 0.72, 95% CI 0.45−1.00, *p* < .001; *I* ^2^ = 45.5%, *p* = .119), but not mixed autoimmune‐autoinflammatory diseases (SMD = 0.46, 95% CI−0.13 to 1.05, *p* = .13; *I* ^2^ = 93.0%, *p* < .001, Figure 9), with lower between‐study variance in the autoinflammatory disease subgroup.

**Figure 10:**
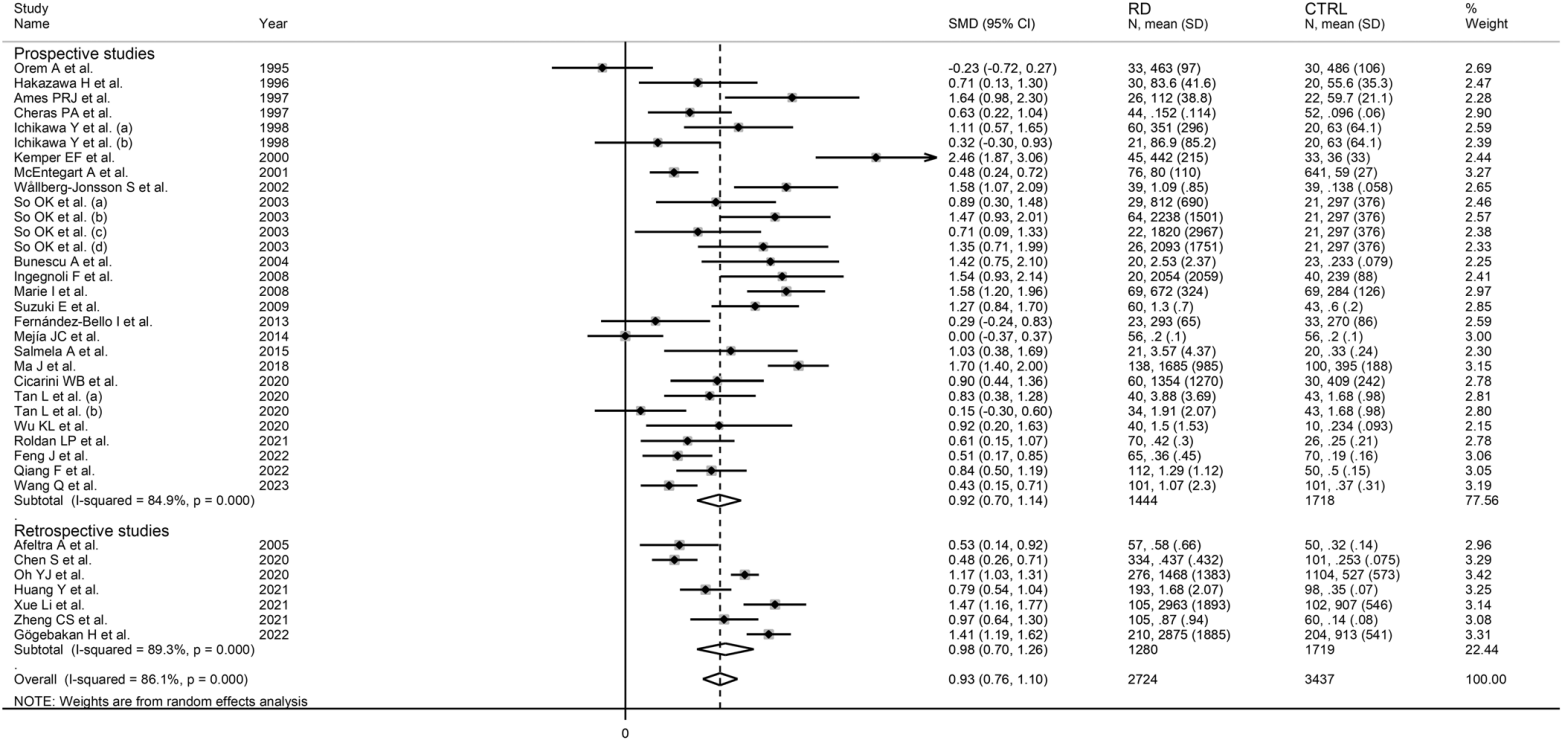
Forest plot of studies investigating d‐dimer concentrations in patients with rheumatic diseases and healthy controls according to study design.

There were no significant differences in the pooled SMD between prospective (SMD = 0.92, 95% CI 0.70−1.14, *p* < .001; *I* ^2^ = 84.9%, *p* < .001) and retrospective studies (SMD = 0.98, 95% CI 0.70−1.26, *p* < .001; *I* ^2^ = 89.3%, *p* < .001; Figure 10).

**Figure 11:**
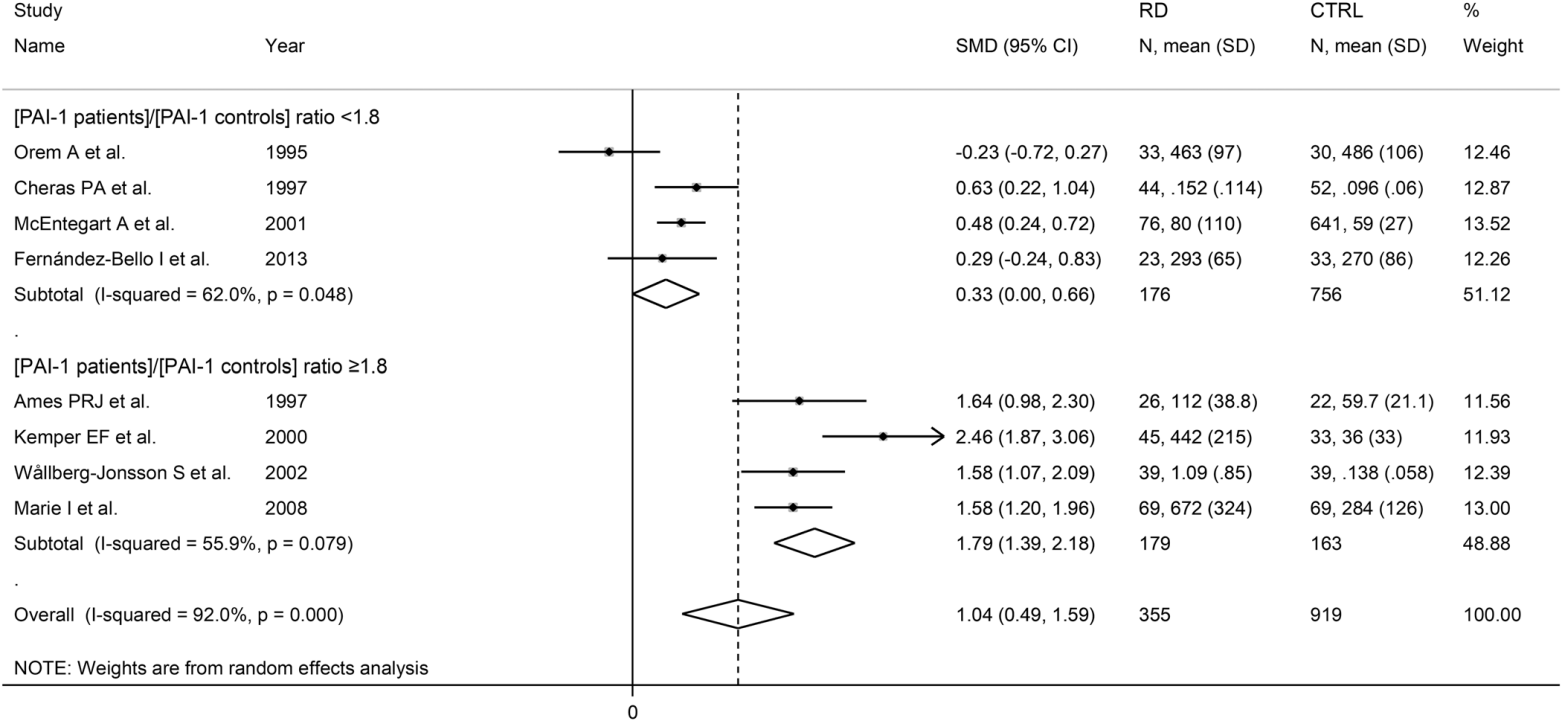
Forest plot of studies investigating d‐dimer concentrations in patients with rheumatic diseases and healthy controls according to the median value of the (PAI‐1 patients)/(PAI‐1 controls) ratio. AI‐1, plasminogen activator inhibitor.

Eight studies also reported serum PAI‐1 concentrations.^62^, ^64^, ^65^, ^67^, ^68^, ^69^, ^74^, ^76^ Following the creation of two subgroups based on the median value of the (PAI‐1 RD patients)/(PAI‐1 controls) ratio, 1.8, there was a significant difference (*p* = .002) in the pooled SMD between studies with ratio <1.8 (SMD = 0.33, 95% CI 0.00−1.66, *p* = .048; *I* ^2^ = 62.0%, *p* = .048) and ratio ≥1.8 (SMD = 1.79, 95% CI 1.39−2.18, *p* < .001; *I* ^2^ = 55.9%, *p* = .079, Figure 11).

**Figure 12:**
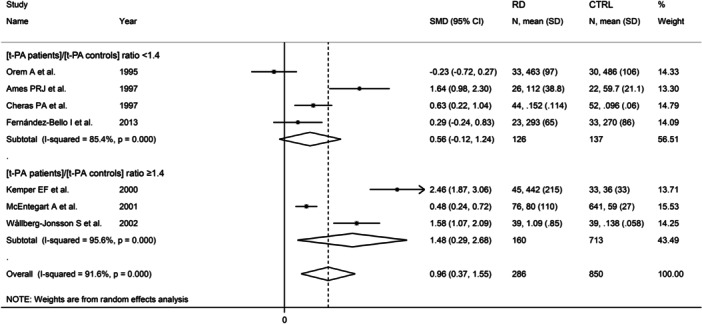
Forest plot of studies investigating d‐dimer concentrations in patients with rheumatic diseases and healthy controls according to the median value of the (t‐PA patients)/(t‐PA controls) ratio. t‐PA, tissue plasminogen activator.

Finally, seven studies also reported serum t‐PA concentrations.^62^, ^64^, ^65^, ^67^, ^68^, ^69^, ^76^ After creating two subgroups based on the median value of the (t‐PA patients)/(t‐PA controls) ratio, 1.4, the pooled SMD was significant when the ratio was ≥1.4 (SMD = 1.58, 95% CI 0.29−2.68, *p* = .015; *I* ^2^ = 95.6%, *p* < .001) but not when it was <1.4 (SMD = 0.56, 95% CI −0.12 to 1.24, *p* = .10; *I* ^2^ = 85.4%, *p* < .001, Figure 12).

The overall level of certainty was upgraded to moderate after considering the low‐moderate risk of bias in all studies (no change), the high but partially explainable heterogeneity (no change), the lack of indirectness (no change), the large effect size (SMD = 0.93, upgrade one level),^93^ and the absence of publication bias (no change).

## 4 DISCUSSION

This systematic review and meta‐analysis showed that, overall, patients with RDs have significantly higher d‐dimer concentrations when compared to healthy controls. However, such elevations differ according to individual RDs and broad RD categories. Specifically, the between‐group differences in d‐dimer progressively decreased in studies of patients with SSc, RA, SpA, AAV, CA, and gout, SLE, OA, and BD. Furthermore, the alterations in d‐dimer concentrations were significant versus controls in patients with autoimmune and autoinflammatory RDs but not in patients with not mixed autoimmune‐autoinflammatory RDs. In meta‐regression and subgroup analyses, significant associations were observed between the effect size of the between‐group differences in d‐dimer concentrations and age, mean RD duration, fibrinogen, CRP, ESR, type of RD, RD subgroup, PAI‐1, and t‐PA. By contrast, no associations were observed with sex, sample size, publication year, use of glucocorticoids and DMARDs, study geographical location, or study design.

The elevations in d‐dimer concentrations in RD patient groups that, unlike RA and SLE, have been relatively less studied in terms of hypercoagulability and risk of VTE, for example, SpA, is likely to foster additional research in this field. Furthermore, despite the known association between BD and DVT in epidemiological and clinical studies,^34^, ^35^, ^36^, ^37^ the effect size of the between‐group differences in d‐dimer concentrations in this patient group was small and nonsignificant compared to other RDs. However, it is important to emphasize that calculating the increased risk of VTE given a particular SMD value is not possible as the SMD is used when studies use different units of measurement for a given variable, in this case d‐dimer concentrations.^94^ Additional research is warranted to investigate whether the relationship between d‐dimer concentrations, hypercoagulability, and risk of VTE is consistent across different RDs, particularly in patients with active disease and/or not receiving optimal pharmacological treatment, or whether other markers of coagulation play a more prominent pathophysiological role in the prevention and management of VTE in specific RDs.

The reported positive associations between the effect size of the between‐group differences in d‐dimer concentrations and age are in line with the known increase in d‐dimer concentrations with advancing age,^95^, ^96^ which has led to the development of age‐adjusted d‐dimer cutoffs to increase the diagnostic performance for VTE.^97^, ^98^ Notably, epidemiological studies investigating the association between RA and VTE have reported an increasing risk with advancing age.^41^, ^99^ However, opposite trends have been observed with SLE.^23^ The positive associations observed between the SMD of d‐dimer concentrations and other markers of thrombosis and coagulation (fibrinogen and PAI‐1) further support a state of hypercoagulability and increased risk of thrombosis in various RDs, whereas the positive associations with established inflammatory biomarkers (CRP and ESR) are in line with the results of studies reporting an increased risk of VTE in RA and SLE patients with increased disease activity.^41^, ^42^, ^43^ The observed significant and negative association between the SMD of d‐dimer concentrations and RD duration is also in line with the results of epidemiological studies in RA patients reporting that the risk of VTE is higher shortly after diagnosis and tends to be stable or decrease afterward.^99^, ^100^, ^101^ However, other studies have reported an increased risk of VTE with longer RD duration.^102^, ^103^ Future studies should investigate whether the alterations in d‐dimer concentrations are subject to temporal variations with longer disease duration, whether these trajectories are similar across different RDs, and whether relatively higher d‐dimer concentrations have a causal relationship with incident VTE in these patients.

Another interesting observation in our subgroup analysis was the absence of significant differences in the pooled SMD between studies conducted on different continents. Although this suggests that the reported elevations in d‐dimer in RDs can be generalized to other ethnic groups, studies in non‐RD populations have consistently reported relatively higher d‐dimer concentrations in African American subjects.^104^, ^105^, ^106^

Strengths of our study include the assessment of d‐dimer concentrations in several types of RDs and broad RD groups, the evaluation of possible associations between the effect size of the between‐group differences in d‐dimer concentrations and various study and patient characteristics, particularly age, CRP, ESR, other markers of coagulation and thrombosis, and RD duration, and a comprehensive assessment of the risk of bias and the certainty of evidence. Furthermore, the results of the meta‐analysis were stable in sensitivity analysis, and no publication bias was observed. The main limitations include the relatively limited number of RDs captured in our systematic search (BD, TA, SSc, OA, RA, SLE, SpA, CA, AAV, gout, AS, and pSS) and the fact that no information was available regarding e causal relationship between d‐dimer alterations and occurrence of DVT/PE.

## 5 CONCLUSIONS

The results of our systematic review and meta‐analysis suggest the presence of significant elevations in d‐dimer concentrations in patients with RDs taken together. However, such alterations depend on specific RD types and categories and are also significantly associated with age, mean RD duration, and other coagulation and inflammatory biomarkers. Additional research is warranted to investigate d‐dimer concentrations in a wider range of RDs and their relationship with disease activity and the occurrence of VTE in this patient group. The results of these studies will determine the true pathophysiological and clinical role of the d‐dimer as a marker of hypercoagulability in patients with RDs and its potential utility in the prevention and management of VTE in these patients.

## AUTHOR CONTRIBUTIONS

**Angelo Zinellu**: Conceptualization; formal analysis; methodology; writing—review and editing. **Arduino A. Mangoni**: Data curation; methodology; project administration; validation; writing—original draft; writing—review and editing.

## CONFLICT OF INTEREST STATEMENT

The authors declare no conflict of interest.

## ETHICS STATEMENT

Ethics approval was not required as this was a systematic review of published studies. Patient consent was not required as this was a systematic review of published studies.

## Supporting information

Supporting information.

## Data Availability

The data supporting the findings of this systematic review and meta‐analysis are available from A. Z. upon reasonable request.
